# Aortic stiffness in the presence of self-limiting and sustained systemic inflammation: comparison of acute myocarditis and chronic inflammatory diseases

**DOI:** 10.1186/1532-429X-16-S1-O78

**Published:** 2014-01-16

**Authors:** Rocio Hinojar, Eduardo Arroyo Ucar, Ning Binti Ngah, Nancy Kuo, David D'Cruz, Nicholas Gaddum, Tobias Schaeffter, Eike Nagel, Valentina Puntmann

**Affiliations:** 1 Cardiovascular Imaging Department, King's College London, London, UK, London, UK

## Background

Aortic stiffness, measured by pulse way velocity (PWV), is an independent predictor of cardiovascular events over and above traditional risk factors. Previous evidence revealed moderately raised PWV in the presence of presence of systemic inflammatory diseases, such as rheumatoid arthritis (RA) and systemic lupus erythematosus (SLE). Changes in aortic stiffness in response to acute systemic inflammation, such as systemic viral myocarditis, remain unknown.

## Methods

Ninety-nine subjects with either clinical diagnosis of acute myocarditis (n = 44) or chronic systemic inflammatory disease (RA and SLE, n = 55) underwent standardized cardiac CMR protocol for the assessment of PWV. Thirty-eight apparently healthy subjects served as control group. Central PWV was obtained by an inplane phase contrast gradient echo sequence with high temporal resolution (120 phases/cardiac cycle) and foot-to-foot measurement.

## Results

Groups were well matched for age, gender and cardiovascular risk factors, with no differences in blood pressure or heart rate between groups. Compared to controls, both patients' groups had significantly raised central PWV (control vs. acute myocarditis vs. systemic inflammation, PWV (m/sec): 5.1 ± 1.0 vs. 8.4 ± 2.4 vs. 8.5 ± 2.6, p < 0.001, with no significant differences between the two groups of patients on post-hoc analysis. We identified significant relationship between PWV and age (controls, r: 0.56; acute myocarditis, r: 0.51; and systemic inflammation, r: 0.3 p < 0.0001 for all), whereas no other functional index showed significant association.

## Conclusions

We demonstrate for the first time that there is increased aortic stiffness in response to self-limiting inflammatory injury, which is comparable in magnitude to sustained systemic inflammation.

## Funding

We would like to acknowledge Department of Health via the National Institute for Health Research (NIHR) comprehensive Biomedical Research Centre award to Guy's & St Thomas' NHS Foundation Trust in partnership with King's College London and King's College Hospital National Health Service Foundation Trust. Dr. Rocio Hinojar was supported by the Fundacion Alfonso Martin Escudero.

